# Universal Restrictions in Reading: What Do French Beginning Readers (Mis)perceive?

**DOI:** 10.3389/fpsyg.2019.02914

**Published:** 2020-01-14

**Authors:** Norbert Maïonchi-Pino, Audrey Carmona, Méghane Tossonian, Ophélie Lucas, Virginie Loiseau, Ludovic Ferrand

**Affiliations:** ^1^Laboratoire de Psychologie Sociale et Cognitive (LAPSCO), CNRS UMR 6024, Université Clermont Auvergne, Clermont-Ferrand, France; ^2^UFR de Sciences Médicales et Pharmaceutiques – Orthophonie, Université de Franche-Comté, Besançon, France

**Keywords:** reading, sonority, markedness, syllable segmentation, illusory conjunctions, French, phonological universals

## Abstract

Despite the many reports that consider statistical distribution to be vitally important in visual identification tasks in children, some recent studies suggest that children do not always rely on statistical properties to help them locate syllable boundaries. Indeed, sonority – a universal phonological element – might be a reliable source for syllable segmentation. More specifically, are children sensitive to a universal phonological sonority-based markedness continuum within the syllable boundaries for segmentation (e.g., from marked, illegal intervocalic clusters, “*jr*,” to unmarked, legal intervocalic clusters, “*rj*”), and how does this sensitivity progress with reading acquisition? To answer these questions, we used the classical illusory conjunction (IC) paradigm. Forty-eight French typically developing children were tested in April (T1), October (T2) and April (T3; 20 children labeled as “good” readers, *M* chronological age at T1 = 81.5 ± 4.0; 20 children labeled as “poor” readers, *M* chronological age at T1 = 80.9 ± 3.4). In this short-term longitudinal study, not only we confirmed that syllable segmentation abilities develop with reading experience and level but the Condition × Sonority interaction revealed for the first time that syllable segmentation in reading may be modulated by phonological sonority-based markedness in the absence or quasi-absence of statistical information, in particular within syllable boundaries; this sensitivity is present at an early age and does not depend on reading level and sonority-unrelated features.

## Introduction

Cross-linguistic evidence indicates that there are regularities across languages. For instance, Consonant-Vowel structures – CV henceforth – are overrepresented across the world’s languages (e.g., Hyman, 2008). By contrast, some coda-onset clusters likewise (i.e., CC) tend to be systematically avoided or underrepresented across syllable boundaries (e.g., /bd/; e.g., Murray and Vennemann, 1983; Vennemann, 1988). But how French beginning readers perceive and segment syllable boundaries they have never read, never heard, or not learnt yet? To address this issue, we focused on *sonority*-based linguistic principles that rule the well-formedness and distinctiveness of phonological sequences (see de Lacy, 2006). More specifically, our question specifically raises the question of whether sonority-based markedness affects and constrains segmentation strategies in the absence or quasi-absence of statistical – distributional – information in visual letter detection.

**FIGURE 1:**
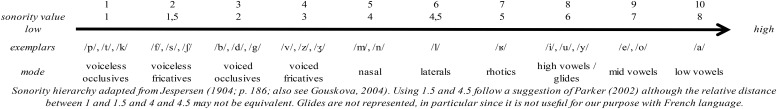
Sonority scale (adapted from Jespersen, 1904; p. 186; also see Gouskova, 2004).

Sonority can be envisaged as a universal, formal, scalar, feature-like phonological element that categorizes all speech sounds into a hierarchical acoustic-phonetic scale. Consonants are ranked from high-sonority phonemes (i.e., from liquid to nasal – labeled *sonorant* –) to low-sonority phonemes (i.e., from fricative to occlusive – labeled *obstruent* –; see Figure 1)^1^. However, sonority remains a controversial linguistic concept, whose nature and origin are a matter of debate (e.g., Clements, 1990, 2006; Ladefoged, 2001; Hayes and Steriade, 2004; Parker, 2008, 2017). Beyond the question of whether sonority is a formally grounded linguistic constraint (i.e., an innate linguistic primitive) or a functionally grounded linguistic constraint derived from speakers’ linguistic experience of the acoustic-phonetic properties of sounds (e.g., Parker, 2017), sonority has different descriptions. Clements (1990, 2006), for example, emphasizes the elusive phonetic correlates in sonority, while Parker (2008) considers that phonological sonority has concrete, quantifiable physical and perceptual properties. Indeed, it has been proposed that sonority is a phonological property of sounds, with their acoustic intensity being the most reliable correlate (e.g., Parker, 2017). In any case, this basic hierarchy of individual sonorities seems to be insufficient, so we now focus on sonority-based linguistic principles to account for restrictions and constraints on the co-occurrence of sounds within and across syllables: the *Sonority Sequencing Principle* (Clements, 1990, 2006) and the *Syllable Contact Law* (e.g., Vennemann, 1988; also see Hooper, 1972; Murray and Vennemann, 1983).

The Sonority Sequencing Principle (Clements, 1990, 2006) is a well-known and extensively studied sonority-based linguistic principle that takes account of the fact that all languages constrain the co-occurrence of sounds within syllables. The Sonority Sequencing Principle depicts syllables’ *Sonority Profile* (SP henceforth), in which universally optimal syllable structures tend to conform to a *Sonority Cycle* that combines and orders sounds. For example, the onset tends to grow toward a maximum sonority at the vowel and fall to a minimum sonority at the coda (e.g., “partir”, *to leave*; although some languages, like Russian or sometimes French and English, violate the sonority generalization made by the Sonority Sequencing Principle, we do not discuss this point here; e.g., Wright, 2004). However, the sonority cycle is not a random phenomenon. According to Clements (1990); also see Selkirk, 1984), an optimal sonority cycle requires the maximum dispersion of individual sonorities between each of the sounds within a C_1_C_2_V syllable, with a minimum rise of at least *x* degrees from C_1_ to C_2_ in a C_1_C_2_ onset cluster (i.e., in reality, three degrees; e.g.,/pni/is better than/pfi/; e.g., Clements, 1990). For instance, within the Optimality Theory framework (e.g., Prince and Smolensky, 2004), which describes a universal, hierarchically ranked set of violable phonological constraints, sonority-based linguistic principles may be interpreted as *markedness constraints* (e.g., *“A syllable contact pair* α.β *is more preferred the greater the decrease in sonority from a coda segment*α *to an onset segment*β”). Thus, in the light of the sonority scale in Figure 1, the least marked (unmarked), and the most well-formed onset cluster will preferentially exhibit a steep rise in SP (e.g., /b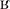/, *s* = + 4). Onset clusters progressively become more marked, and less well-formed (marked) as the SP decreases from high-rise to low-rise (e.g., /ml/, *s* = + 1), then on to plateau SP (e.g., /bd/, *s* = 0), low-fall SP (e.g., /sp/, *s* = −1), and high-fall SP (e.g., /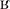
b/, *s* = −4). As markedness increases, well-formedness decreases from high-rise SP to high-fall SP. However, this point concerns the syllable structures themselves, whereas we are interested in the syllable boundaries here.

**FIGURE 2:**
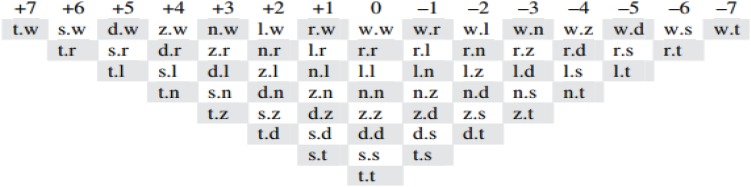
Stratified relational hierarchy of coda and onset proposed within the SYLLABLE CONTACT constraint, where “r” stands for rhotics, “t” for voiceless occlusives, “d” for voiced occlusives, “z” for voiced fricatives, “s” for voiceless fricatives, “n” for nasals, “l” for laterals, and “w” for glides (adapted from Gouskova, 2004).

To cope with the restriction of the Sonority Sequencing Principle to the internal structure of syllables, we refer to the Syllable Contact Law (e.g., Murray and Vennemann, 1983; Vennemann, 1988) that predicts a gradient preference for universally optimal syllable contact as follows: “*A syllable contact pair*α.β *is more preferred the greater the increase in consonantal strength from a coda segment*α *to an onset segment*β.” Consonantal strength has thereafter been reinterpreted in terms of sonority to describe an optimal syllable contact between two adjacent segments (e.g., Clements, 1990). In other words, the Syllable Contact Law categorically prohibits a sonority rise across syllable boundaries (e.g., “mar.teau”, *hammer* > “pa.tron”, *boss*, not “pat.ron”; the dot represents the expected location of the syllable boundary). For instance, implemented within the Optimality Theory, the Syllable Contact Law shapes a categorical, hierarchically ranked set of violable markedness constraints to describe optimal syllable contacts, due to which *“sonority should not rise across a syllable boundary”* (e.g., Holt, 2004). Following this, Gouskova’s (2004) proposal is of particular interest for our study. Gouskova (2004) proposed a more sophisticated and fine-grained gradient-based formalization of the syllable contact constraint implemented within Optimality Theory: a syllable contact should be envisaged as a stratified relational hierarchy that determines the well-formedness of a coda or onset not in isolation but in relation to the adjacent onset or coda, respectively (see Figure 2). Gouskova’s (2004) collapses the individual sonority in coda position and the individual sonority in onset position into a single scale (the coda tends to be the more sonorous consonant, while the onset tends to be the less sonorous consonant; see Jespersen, 1904; Prince and Smolensky, 2004; Zec, 2007). The least marked, the most well-formed, the most harmonic SP preferentially exhibits a steep fall from the coda (C_1_) to the onset (C_2_). Consequently, the SP becomes more marked, less well-formed, and less harmonic as the coda becomes less sonorous and the onset becomes more sonorous (e.g., /lb/ > /bd/ > /bl/; > stands for “preferred over”^2^). It is interesting to note that any syllable contact provides a *Sonority Distance* which is not sensitive to the type of consonant (i.e., fricative, nasal, obstruent, etc.); the way a C_1_C_2_ cluster is processed is therefore predicted not by the type of consonants but only by the sonority distance. Hence, “w.z”, “r.d”, “l.s”, and “n.t” belong to the same stratum and are theoretically equivalent since these clusters share the same sonority distance (i.e., −4; see Figure 2). The sonority contact scale in Figure 2 indicates that a syllable contact preferentially exhibits a steep fall in SP across syllable boundaries (unmarked, most harmonic; e.g., high-fall SP such as/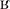
b/, *s* = −4); syllable contacts progressively become more marked, and less well-formed (marked, less harmonic) as the SP increases across syllable boundaries from high-fall SP to low-fall SP (e.g., /df/, *s* = −1), then on to plateau SP (i.e., null distance; e.g., /bd/, *s* = 0), low-rise SP (e.g., /ml/, *s* = + 1), and high-rise SP (e.g., /b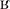/, *s* = + 4). The markedness pattern across syllable boundaries is therefore the complete opposite of the markedness pattern in onset clusters. For the sake of clarity and consistency, we speak of a *phonological sonority-based markedness* to designate the SP in either onset or intervocalic position.

Investigating the influence of sonority-based contact between adjacent syllables on syllable location and segmentation strategies is particularly relevant in French, which is defined as a syllable-timed language with clear-cut syllable boundaries and mostly polysyllabic words (e.g., Kaye and Lowenstamm, 1984; i.e., >90%; Manulex database^3^; Lété et al., 2004). French also permits both simple and complex syllable structures as well as intervocalic C_1_C_2_ clusters (i.e., 60% of CV structures vs. 17% of CVC structures vs. 14% of CCV structures; e.g., Léon, 2007; Manulex-infra database; Peereman et al., 2007). Research conducted over the last 20 years has suggested that syllables are salient perceptual and segmental units in French newborns and preliterate children (e.g., Bertoncini et al., 1988; Duncan et al., 2006; Nazzi et al., 2006; Goslin and Floccia, 2007). They are also fundamental reading units in French children who are learning to read (e.g., Maïonchi-Pino et al., 2012a, b; Doignon-Camus and Zagar, 2014). To better understand why syllables are crucial reading units, it is worth noting that learning to read follows a small-to-large developmental sequence that progresses from grapho-phonemic processing to grapho-syllabic processing (e.g., Ehri, 2005). This hypothesis supports the idea that first children have to learn the grapheme-to-phoneme correspondences to progressively *consolidate* and *unitize* them into larger units. This is particularly suitable since grapho-syllabic processing requires “f*ewer connections to secure the word in memory*” (Ehri, 2005, p. 175), while grapho-phonemic processing requires considerable attentional resources due to the sequential left-to-right processing of each unit in turn.

To explain the developmental course and the role of syllable-sized units in reading, most research tends to reduce the syllable effects to the question of statistical properties. Of the previous studies that have specifically addressed the question of the developmental course of syllable segmentation in French children, those by Colé et al. (1999) and Maïonchi-Pino et al. (2010) have suggested that the use of syllable-sized units depends on:

(1) Reading level (“good” readers use syllables earlier and better than “poor” readers);
(2) Reading instruction (grapho-syllabic processing comes after grapho-phonemic processing through a process of progressive bidirectional consolidation after 6 months of reading instruction, at least in “good” readers);
(3) Statistical properties (after 6 months of reading instruction, children use syllables for high-frequency syllables, while phoneme-sized units are used for low-frequency syllables, before the use of syllable-sized units becomes generalized after 2.5 years of reading instruction; e.g., Maïonchi-Pino et al., 2010).

Consequently, a vast body of evidence provided robust arguments on the role of statistical and distributional properties with either contrasted statistical distribution effects – e.g., the *bigram trough* –, or effects that contradict or modulate syllable effects – e.g., inhibitory vs. facilitatory effects – (e.g., Doignon and Zagar, 2006; Chetail and Mathey, 2009a, b, 2013; Maïonchi-Pino et al., 2010; Chetail, 2015). The widely accepted and well-documented observation that children efficiently develop robust representations of the statistical orthographic and phonological distribution of their language could be confronted with the role of phonological universals. To date, the literature provides no extensive and clear-cut answers. Critically, phonological universals such as sonority have not been a clear focus of study, except in the case of research into speech production and perception, despite the fact that it might be a credible candidate that co-contributes with or even compensates for statistical properties in silent reading (e.g., in speech perception in English, Russian, and Korean, Berent et al., 2007, 2008, 2011, 2012a; in Spanish, Berent et al., 2012b; in French, Maïonchi-Pino et al., 2013; in Mandarin Chinese, Zhao and Berent, 2016). Given that sensitivity to sonority-based constraints might be available at an early age and independently of linguistic experience and that it might also contribute to language acquisition (e.g., Gómez et al., 2014), current studies should examine how and when phonological sonority-based markedness impacts reading. Our prediction is that French children might refer to non-statistical properties when implementing syllable location and segmentation strategies in silent reading by taking advantage of their sensitivity to sonority-based constraints (e.g., Fabre and Bedoin, 2003; Maïonchi-Pino et al., 2012a, b, 2015).

Empirical evidence regarding sonority in reading-related French studies remains scarce (but see Sprenger-Charolles and Siegel, 1997; Marouby-Terriou and Denhière, 2002; Maïonchi-Pino et al., 2012a, b, 2015). For instance, studies of reading aloud in preliterate and literate French children found that these children often reduced complex CVC or CCV syllable structures into simple CV syllables, thus indicating a preference to maximize sonority distance and eliminate sonorant consonants (e.g., “tru” → “tu”; “bar” → “ba”; e.g., Sprenger-Charolles and Siegel, 1997; Bastien-Toniazzo et al., 1999; Gnanadesikan, 2004). Crucially, these studies are limited to attested SPs, that is to say those with quantifiable orthographic and phonological statistical properties (e.g., Fabre and Bedoin, 2003; Maïonchi-Pino et al., 2012a, b). In the case of French readers, it would be particularly important to examine syllable location and segmentation strategies. Although SPs might be crucial for syllable segmentation strategies in French children, there is no clear dissociation between what is due to SPs – not considered as markedness patterns in previous studies (e.g., Fabre and Bedoin, 2003; Maïonchi-Pino et al., 2012a, b) – and what derives from statistical properties or from linguistic features that might account for the universal sensitivity to C_1_C_2_ clusters within syllable boundaries.

Given that we wanted to suppress the orthographic and phonological information to potentiate the sonority-based effects, we decided to use the IC paradigm – which does not tap into the lexical processes – in combination with pseudowords, since these limit the top-down lexical processes (IC paradigm henceforth; e.g., Prinzmetal et al., 1986, 1991). Since 2003, the IC paradigm has been the subject of renewed interest for investigating whether syllables are activated quickly and automatically, and determining how the statistical (orthographic and/or phonological) distribution influences segmentation strategies in French typically developing and dyslexic children (e.g., Fabre and Bedoin, 2003; Doignon and Zagar, 2006; Maïonchi-Pino et al., 2012a, b; Doignon-Camus et al., 2013; Doignon-Camus and Zagar, 2014). An IC is a misperception of the color of a target-letter under conditions involving high attentional and perceptual demands. Two IC patterns coexist. For instance, in the case of a word like ANVIL presented either as ANVil or ANvil (where upper- and lower-case letters represent two different colors) there are: (1) ICs that preserve the syllable boundary (i.e., the participant reports that the target-letter “V” is the same color as “il” in “AN.Vil”; the dot represents the syllable boundary); and (2) ICs that violate the syllable boundary (i.e., the participant reports that the target-letter “v” is the same color as “AN” in “AN.vil”). If children really perceive syllable units in pseudowords, preservation ICs would exceed violation ICs. Based on this color misperception, which is thought to be caused by automatic syllable extraction, researchers have focused on a specific statistical argument – the *bigram trough hypothesis* – to explain syllable segmentation strategies (e.g., Seidenberg, 1987). The *bigram trough hypothesis* refers to the statistical distribution of letter co-occurrences, which follows language-specific phonotactic restrictions that govern how, and how frequently, letters occur and co-occur in specific positions. The letter co-occurrences that straddle syllable boundaries (e.g., the bigram NV in ANVIL) are of lower frequency than cluster co-occurrences that constitute syllable-sized units that precede or follow the syllable boundaries (e.g., AN and VI) and act as a *powerful* statistical cue that underlies syllable location and segmentation. However, previous research has confirmed that orthographic statistical properties do not occur in isolation (e.g., in Spanish, Conrad et al., 2009(in English, Rapp, 1992; Muncer et al., 2014). For instance, in French, Doignon and Zagar (2006) and Doignon-Camus and Zagar (2014) found that, from the age of six, French children’s response patterns indicated more preservation ICs than violation ICs when the orthographic and phonological boundaries matched (e.g., “BI/MIR” and “RON/TA”; the slash indicates that the orthographic boundary coincides with the phonological boundary) but that the response patterns indicated more violation ICs than preservation ICs when the orthographic and phonological boundaries mismatched (e.g., “RO/N^∗^ER” and “BI^∗^M/BU”; the ^∗^ indicates the orthographic boundary, whereas the slash indicates the phonological boundary). Taken together, these results have led some authors to conclude that children, including those suffering from developmental dyslexia, are sensitive to – and have developed knowledge about – orthographic statistical distribution, whereas syllable boundary location and segmentation seem to depend strictly on the overlap between statistical properties. For instance, Maïonchi-Pino et al. (2012a, b) found that French children have clear-cut sonority-driven syllable segmentation strategies, in particular in the case of the optimal ‘sonorant coda – obstruent onset’ SPs. As mentioned earlier, their conclusions unfortunately stem from some attested C_1_C_2_ with quantifiable orthographic and phonological statistical properties analyzed *a posteriori*. Although their studies concluded that neither phonological properties nor orthographic properties provided a straightforward account for sonority-modulated syllable segmentation, there was no clear dissociation between attested vs. unattested intervocalic clusters, while children could have inferred the well-formedness of a C_1_C_2_ cluster across syllables from its existence in real French words.

To further determine the influence of phonological sonority-based markedness across the syllable boundaries in the absence or quasi-absence of statistical information, whether orthographic or phonological, and to track developmental changes in the segmentation strategies of children who are learning to read, we used the IC paradigm to expose French “good” and “poor” readers to target-letters that had to be detected within the syllable boundaries of two-colored pseudowords. These trials were performed in April (a), October and April (b). We decided to start after 8 months of learning to read [April (a)] since previous studies showed that syllable-based effects in French are found from February in good readers (e.g., Colé et al., 1999). The orthographic and phonological statistical properties were null or quasi-null around the syllable boundary, while sonority-based markedness was manipulated along a continuum from marked, ill-formed SPs to unmarked, well-formed SPs. Our main predictions were:

(1) If phonological representations are modeled on the basis of sonority-based markedness constraints, we predict an early and robust sensitivity to the well-formedness of the SPs (i.e., color of the target-letter assigned preferentially to high-fall SPs than to high-rise SPs) as of April (T1) in “good” and “poor” readers;
(2) If segmentation strategies require sensitivity to sonority-based markedness even in the absence or quasi-absence of statistical properties, once syllable segmentation becomes available in the developmental time course, we expect that preservation ICs should increase while violation ICs should decrease, provided the well-formedness of the SPs;
(3) If syllable segmentation follows a progressive developmental course coupled with the difficulties to face unattested phonological clusters, we expect that neither “good” nor “poor” readers should exhibit syllable effects in April (a), whereas these should emerge as of October and be more pronounced in “good” readers (more preservation ICs in “good” readers than in “poor” readers).”

## Materials and Methods

### Participants

Forty-eight French typically developing children from an urban elementary school were tested in April (T1), October (T2), and April (T3). All the children were from a medium socio-economic background, were monolingual native speakers of French, right-handed (+0.80 and +1 right-handedness scores were measured with the Edinburgh Handedness Inventory; Oldfield, 1971), and had normal or corrected-to-normal vision and hearing. At T1, all the children were first-graders who had been taught to read for 8 months using a mixture of analytical GPC and global procedures. We used a 20-min French standardized age-based word reading test at T1 to categorize them as “good” and “poor” readers (TIMÉ 2; Écalle, 2003). Children with a reading level of between −2 and 6 months were considered to be “good readers” (*N* = 22), while children with a reading level of between −3 and −9 months were considered to be “poor readers” (*N* = 26). TIMÉ 2 was also used at T2 and T3 and eight children who switched from good-to-poor or poor-to-good between T1–T2 (*N* = 3) or T2–T3 (*N* = 4) or who repeated a year (*N* = 1) were excluded from the analyses. Forty children were therefore included in the analyses (7 boys and 13 girls labeled as “good” readers; 11 boys and 9 girls labeled as “poor” readers). Descriptive and statistical data are provided in Table 1. The children neither exhibited reading or intellectual disorders nor had reading levels that differed by more than 9 months from their chronological age between T1 and T3 (*M* difference = 2.7 months ± 2.2, *min* = −9, *max* = 6). All the children participated after their parents had completed and signed an informed consent form. This research was approved by the Regional School Management Office and the Regional Ethics Committee.

**TABLE 1:** Descriptive data for the “good” and “poor” readers at T1, T2, and T3.

	**Time 1**		**Time 2**		**Time 3**			
	**Chronological age**	**Reading level**	**Chronological age**	**Reading level**	**Chronological age**	**Reading level**	***Variation T1-T3***	
							**Chronological age**	**Reading level**
Good readers	81.5 (4.0)	84.2 (3.1)	87.5 (4.0)	90.4 (3.3)	93.5 (4.0)	96.3 (3.4)	+12,0	+12.1
Poor readers	80.9 (3.4)	77.5 (3.4)	86.9 (3.4)	82.7 (3.3)	92.9 (3.4)	88.4 (3.6)	+12,0	+10.9
*Mean*	81.2	80.9^∗∗∗^	87.2	86.6^∗∗∗^	93.2	92.4^∗∗∗^		

*^∗∗∗^Indicates a significant 0.0001 *p-*value; ages are in months, standard deviation in brackets.*

### Material

Thirty-five six-letter disyllabic pseudowords were created. The three initial letters of these words had a VCC structure and a final schwa-like vowel (e.g., VCCVCV, *ojrule*). All the pseudowords had three consonants and three vowels which had regular spelling-to-sound correspondences^4^. All the disyllabic pseudowords had an intervocalic C_1_C_2_ cluster (i.e., V_1_C_1_C_2_V_2_C_3_V_3_). All the intervocalic C_1_C_2_ clusters were considered unattested both in word-initial position and as syllable-initial structures in French (e.g., Dell, 1995; Peereman et al., 2007). Homorganic consonants^5^ and voicing differences were avoided within the C_1_C_2_ clusters (regressive/progressive voicing assimilation). We used the following homorganic consonant classification: labial (i.e., /p/,/b/,/f/,/v/, and/m/), coronal (i.e., /n/,/t/,/d/,/l/,/s/,/z/,/∫/, and/Ȝ/), and dorsal (i.e., /k/,/g/, and/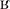/). C_1_ and C_2_ could have a different manner of articulation (i.e., obstruent, fricative, nasal, or liquid; see Appendix).

Regarding the unattested status of the C_1_C_2_ clusters in word-initial position, we predicted syllable segmentation between the C_1_ and C_2_ consonants (e.g., Spencer, 1996). Theoretically speaking, three segmentations were possible: V_1_.C_1_C_2_V_2_C_3_V_3_, V_1_C_1_.C_2_V_2_C_3_V_3_, or V_1_C_1_C_2_.V_2_C_3_V_3_. Whatever the syllable segmentation, and regardless of whether the segmentation respected or violated the phonotactic restrictions of French, the initial syllable structure was among the rarest in French (i.e., V, 8%; VC, 1.9%, and VCC, 0.5%; Léon, 2007). To keep the statistical properties of the orthographic and phonological components to null or quasi-null values for the V_1_C_1_C_2_V_2_C_3_ pattern, we referred to the Manulex-infra database (Peereman et al., 2007), which provides grade-level sublexical frequencies for French elementary-school readers, and the Surface database computed from the Lexique 2 database (New et al., 2004), which provides the distributional sublexical frequencies for French adult readers. Using the Manulex-infra database, we calculated the mean U_1_–U_2_ frequency, which designates the mean frequency computed for first and second grades, for the different sublexical structures (orthographic and phonological) of the V_1_C_1_C_2_V_2_C_3_V_3_ patterns, while the Surface database provided a confirmation of the exact mean positional frequencies. The exclusive use of the Manulex-infra database and Surface database ensured there was no clear bigram trough. Descriptive data are reported in Table 2.

**TABLE 2:** Mean frequencies for the different positions of the orthographic and phonological constituents in the V_1_C_1_C_2_V_2_C_3_V_3_ pseudowords calculated with the Manulex-infra database (Peereman et al., 2007) and the Surface database (New et al., 2004).

			**Sonority Profiles**					
	**Frequency**	**Database**	**High-Fall SPs**	**Low-Fall SPs**	**Plateau SPs**	**Low-Rise SPs**	**High-Rise SPs**	***Mean***
Orthographic frequencies	Frequencies for the V_1_C_1_ bigrams (inital position)	Manulex-infra	11.4	0.0	3.9	2.5	10.6	5.7
		Surface	73.6	0.4	16.3	19.1	9.3	23.8
	Frequencies for the C_1_C_2_ bigrams (that straddle the syllable boundary; all the C_1_C_2_ bigrams have a null frequency in initial position, *M* = 0 ± 0)	Manulex-infra	16.4	0.1	0.4	0.7	6.2	4.8
		Surface	11.9	0.1	0.0	7.9	62.9	16.5
	Frequencies for the C_2_V_2_ bigrams (that follow the syllable boundary; the C_2_V_2_ bigrams have equivalent frequencies in initial position)	Manulex-infra	67.7	93.4	82.1	99.8	121.1	92.8
		Surface	72.2	84.3	82.8	69.2	59.0	73.5
	Frequencies for the V_1_C_1_C_2_ trigrams	Manulex-infra	0.0	0.0	0.0	0.0	0.0	0.0
		Surface	0.0	0.0	0.0	0.0	0.0	0.0
	Frequencies for the C_1_C_2_V_2_ trigrams	Manulex-infra	0.0	0.0	0.0	0.0	0.0	0.0
		Surface	0.0	0.0	0.0	0.0	0.0	0.0
	Frequencies for the C_2_V_2_C_3_ trigrams	Manulex-infra	0.0	0.0	0.0	0.0	0.0	0.0
		Surface	0.0	0.0	0.0	0.0	0.0	0.0
	Frequency distance between V_1_C_1_ bigrams and C_1_C_2_ bigrams	Manulex-infra	0.3	0.1	–6.1	–1.7	–4.4	–2.4
	Frequency distance between C_1_C_2_ bigrams and C_2_V_3_ bigrams		99.0	137.6	58.1	84.7	132.1	102.3
Phonological frequencies	Frequencies for the V_1_C_1_ syllables	Manulex-infra	25.6	4.1	4.7	8.6	8.0	10.2
	Frequencies for the V_1_C_1_C_2_ syllables		0.0	0.0	0.0	0.0	0.0	0.0
	Frequencies for the C_2_V_2_ syllables		239.9	291.7	407.1	450.6	752.4	428.3
	Frequencies for the C_2_V_2_C_3_ syllables		0.0	0.3	9.9	0.0	0.7	2.2

*Frequencies are given in occurrences per million.*

Unattested intervocalic C_1_C_2_ clusters were divided into five SPs (7 pseudowords × 5): high-fall (e.g., *lb*; *s* = −4, −3 or −2), low-fall (e.g., *fk*; *s* = −1), plateau (e.g., *kp*; *s* = 0), low-rise (e.g., *zm*; *s* = + 1), and high-rise (e.g., *zr*; *s* = + 2 or + 3). Intervocalic cluster markedness fell from high-rise SPs (the most marked and most ill-formed) to high-fall SPs (the least marked and most well-formed). Each SP comprised seven different C_1_C_2_ clusters.

Two colors (red and blue) were assigned to two different segments of a pseudoword. No two segments ever had the same color. This resulted in two experimental conditions. In the color-syllable compatibility condition, the color segmentation matched the expected syllable segmentation (e.g., UL.byre), whereas in the color-syllable incompatibility condition, the color segmentation mismatched the expected syllable segmentation since segmentation occurred either before (e.g., Ul.byre) or after (e.g., UL.Byre) the syllable boundary. The target-letters to be detected were either the second or the third letter within the syllable boundary and were always at the border of the colored segments in order to prevent lateral masking (e.g., L or b in ULbyre; l in Ulbyre; B in ULByre). Each pseudoword was repeated four times (*N* = 140): twice in the color-syllable compatibility condition (i.e., L in ULbyre and b in ULbyre) and twice in the color-syllable incompatibility condition (i.e., l in Ulbyre and B in ULByre). In the color-syllable compatibility condition, since color and syllable boundaries matched only *violation ICs* occurred when the color of “L” or “b” in “ULbyre” was misperceived as being the same color as “byre” or “UL”, respectively. In the color-syllable incompatibility condition, since color and syllable boundaries mismatched only *preservation ICs* occurred when the color of “l” in “Ulbyre” was misperceived as being the same color as “U” or when the color of “B” in “ULByre” was misperceived as being the same color as “yre”.

### Procedure

The children each individually completed a single task (*M* duration = 16 min ± 2). The script was designed, compiled and run with E-Prime 2 Professional (Schneider et al., 2002) on Sony X-series laptop computers running under Windows 7. The children sat 57 cm from the screen in a silent room. Each trial proceeded as follows: a vertically centered green square (0.82° of visual angle) was displayed for 250 ms. This was then replaced by a black uppercase target-letter which was displayed in the center of the screen for 1,250 ms (0.41° of visual angle). This was followed by a 250-ms white screen which was then replaced by a two-colored pseudoword, which covered 2.46° of visual angle, flashed up at a visual angle of 1.14° from the screen. A black question mark (?) then appeared in the center of the screen, where it remained until the child responded (after 5,000 ms, a warning message indicated the absence of response and this was considered to be an IC; *N* = 44; 0.26%. The next trial then started. The next trial followed after a 750-ms interval.

The duration of the flashed two-colored pseudowords was adjusted for each child in order to maintain an average IC rate of 20–25% throughout the task. The duration calculation involved a two-step adjustment. First, each child was trained with a practice list on 24 trials and received corrective feedback (no feedback was given in the experimental lists). The initial exposure duration in the practice list was set to 350 ms (21 refresh cycles of 16.67 ms). The exposure duration was adjusted every three trials in the practice list in decrements and increments of one refresh cycle. Second, the mean exposure duration for each child at each time was used to set the initial exposure duration in the first experimental list (“good” readers, *M* = 309 ms ± 31; range = 300–315; “poor” readers, *M* = 314 ms ± 28; range = 308–325). For the experimental lists, the increment/decrement procedure was the same as for the practice list (“good” readers, *M* = 281 ms ± 28; range = 266–292; “poor” readers, *M* = 297 ms ± 30; range = 288–304).

The pseudowords in the syllable-color compatibility and syllable-color incompatibility conditions were counterbalanced across five experimental lists that were separated by pauses. The distribution of the pseudowords was pseudo-randomized, whereas the order of their presentation was randomized. To avoid decision bias in the experimental trials, we inserted two fillers after each pause (*N* = 10), i.e., at the beginning of each experimental list, and the corresponding results were not included in the statistical analysis. The children were instructed to report the color of the target-letter in the flashed pseudowords as quickly and accurately as possible. They had to press on the “blue” or “red” response keys (“r” and “n” keys, respectively). The software automatically recorded ICs and response times.

## Results

We used Statistica 8.0 software^®^ (StatSoft^®^, 2011) to run a 3 × 2 × 5 × 2 mixed-design repeated-measures analysis of variance (ANOVA) by subject (*F*1) and by item (*F*2) for the ICs (±16.31% of the data), with Time (T1; T2; T3), Condition (compatibility; incompatibility), Target-letter (second letter; third letter) and SP (high-fall; low-fall; plateau; low-rise; high-rise) as within-subject factors and Group (good readers; poor readers) as between-subject factor. Correct response times were trimmed (i.e., for each subject, response times that deviated by ± two standard deviations from the mean were considered as ICs, 0.96% of the data). Descriptive data are summarized in Table 3.

**TABLE 3:** Mean illusory conjunction rate for the Group × Time × Condition × Sonority profile × Target-letter mixed-design.

	**Time 1**				**Time 2**				**Time 3**			
	**Color-syllable compatibility**		**Color-syllable incompatibility**		**Color-syllable compatibility**		**Color-syllable incompatibility**		**Color-syllable compatibility**		**Color-syllable incompatibility**	
	**Second letter**	**Third letter**	**Second letter**	**Third letter**	**Second letter**	**Third letter**	**Second letter**	**Third letter**	**Second letter**	**Third letter**	**Second letter**	**Third letter**
**Good readers**												
High-fall	10.0	10.7	26.4	22.9	7.1	6.4	30.7	30.0	7.1	6.4	27.1	26.4
Low-fall	12.9	12.1	25.0	22.9	10.0	8.6	25.7	24.3	7.9	7.9	25.0	23.6
Plateau	18.6	17.1	17.1	15.7	13.6	11.4	22.9	20.7	14.3	12.9	23.6	22.9
Low-rise	23.6	19.3	12.9	13.6	20.0	16.4	11.4	15.0	18.6	15.0	12.1	17.1
High-rise	22.1	19.3	12.1	14.3	23.6	17.9	10.0	14.3	19.3	13.6	10.7	16.4
*Mean*	17.4	15.7	18.7	17.9	14.9	12.1	20.1	20.9	13.4	11.1	19.7	21.3
**Poor readers**												
High-fall	10.0	12.9	21.4	18.6	11.4	10.7	20.0	17.9	9.3	7.9	22.1	20.7
Low-fall	11.4	13.6	21.4	20.7	10.7	10.0	20.0	18.6	8.6	10.0	20.7	20.0
Plateau	15.0	15.7	15.7	13.6	13.6	12.1	18.6	16.4	14.3	14.3	20.0	19.3
Low-rise	19.3	16.4	12.1	13.6	19.3	16.4	11.4	15.0	20.7	19.3	12.1	15.7
High-rise	22.1	18.6	10.0	12.1	21.4	18.6	10.0	14.3	20.7	18.6	11.4	14.3
*Mean*	15.6	15.4	16.1	15.7	15.3	13.6	16.0	16.4	14.7	14.0	17.3	18.0

*Response are given in percentage (%). Errors in the color-syllable compatibility correspond to violation ICs; errors in the color-syllable incompatibility correspond to preservation ICs.*

### Discrimination Sensitivity Threshold and Decision Criterion Analysis

We referred to *signal detection theory* to assess both the *d’* value (discrimination sensitivity threshold) and the β value (decision criterion), which highlights the possible bias toward the (mis)perception of the target-letters within the two-colored pseudowords (e.g., Macmillan and Creelman, 2005). The *d’* value reflects the correct detection of the color of the target-letters within the two-colored pseudowords. None of the participants had a *d’* = 0 ± 5% (*d’* = 0 ± 5% refers to *random responses* embedded between 47.5 and 52.5%). We observed that the *d’* criterion fluctuated significantly as a function of Group, *F*(1, 114) = 4.77, *p* < 0.03, η^2^_p_ = 0.04 (“good” readers, *M* = 2.183; “poor” readers, *M* = 2.258). The distribution for the *d’* criterion was harmonic around 2 (*M* = 2.221 ± 0.196; *min* = 1.606, *max* = 2.757), indicating that detection was difficult, with a low to moderate sensitivity threshold. The β criterion reflects the possible bias toward syllable or non-syllable segmentation, and we observed that the β criterion varied significantly as a function of both Time, *F*(2,114) = 14.12, *p* < 0.0001, η^2^_p_ = 0.20 (T1, *M* = 1.246; T2, *M* = 1.081; T3, *M* = 1.012) and Group, *F*(1,114) = 28.04, *p* < 0.0001, η^2^_p_ = 0.20 (“good” readers, *M* = 1.016; “poor” readers, *M* = 1.211). The distribution for the β criterion started above 1 and tended to 1 and even to below 1, thus suggesting a progressive bias toward syllable segmentation (*M* = 1.112 ± 0.237; *min* = 0.515, *max* = 1.762).

### Illusory Conjunctions Analysis

**FIGURE 3:**
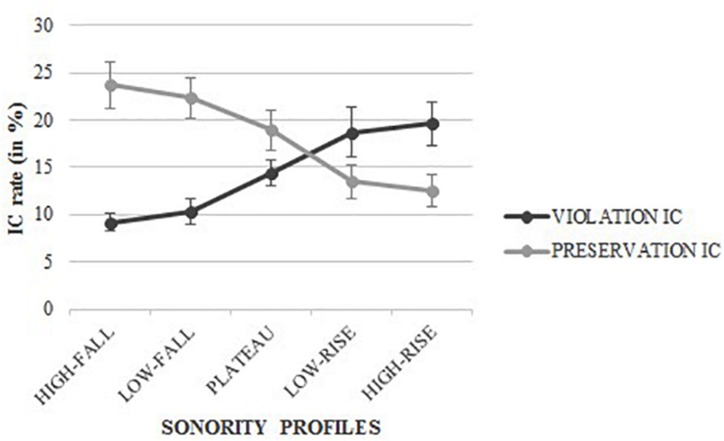
Illusory conjunction rate (IC) in percentage (%) for the Condition × Sonority interaction.

**FIGURE 4:**
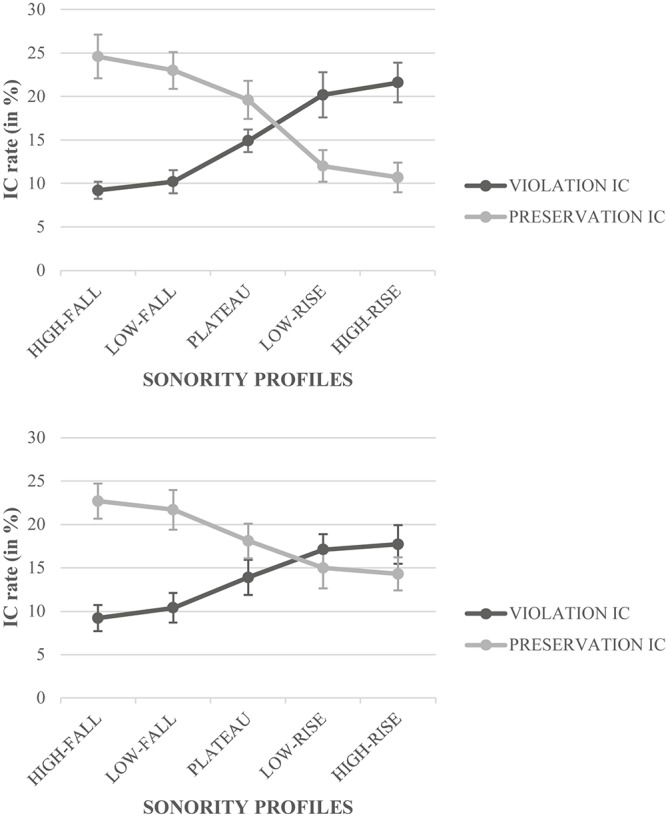
Illusory conjunction rate (IC) in percentage (%) for the Condition × Sonority × Target-letter interaction as a function of the Target-letter (**upper panel**, the second letter; **lower panel**, the third letter).

**FIGURE 5:**
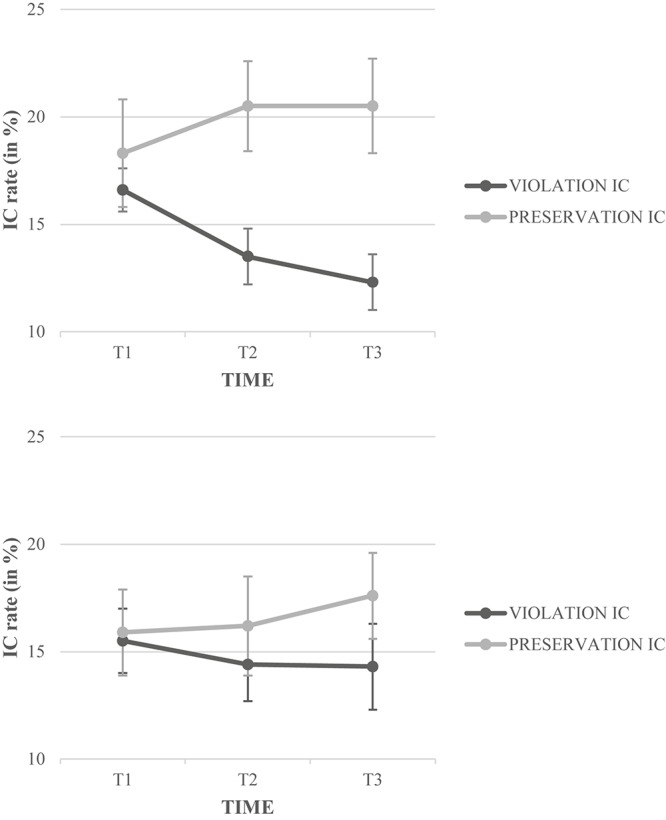
Illusory conjunction rate (IC) in percentage (%) for the Condition × Time × Group interaction as a function of the Group (**upper panel**, good readers; **lower panel**, poor readers).

We ran an ANOVA on the proportion of ICs (16.31% of the data). All the Fisher’s LSD *post hoc* tests have been conducted on the *F*1 only. The results showed a significant main effect of Condition, *F*1(1,38) = 49.22, *p* < 0.0001, η^2^_p_ = 0.56, *F*2(1,120) = 24.71, *p* < 0.0001, η^2^_p_ = 0.17, with the children producing significantly more preservation ICs (18.18%) than violation ICs (14.44%). The Condition × Group interaction was significant, *F*1(1,38) = 12.78, *p* < 0.001, η^2^_p_ = 0.25, *F*2(1,120) = 5.62, *p* < 0.02, η^2^_p_ = 0.04; a Fisher’s LSD *post hoc* test (Bonferroni’s adjusted α level for significance, *p* < 0.008) indicated that good readers outperformed poor readers on the preservation ICs only, while both good and poor readers produced more preservation ICs than violation ICs. The Condition × Sonority interaction was significant (Figure 3), *F*1(4, 152) = 39.32, *p* < 0.0001, η^2^_p_ = 0.51, *F*2(4,120) = 7.53, *p* < 0.001, η^2^_p_ = 0.17; a Fisher’s LSD *post hoc* test (Bonferroni’s adjusted α level for significance, *p* < 0.001) revealed that preservation ICs increased continuously from high-rise SPs to high-fall SPs, while violation ICs decreased monotonically from high-rise SPs to high-fall SPs. Additionally, the Condition × Sonority × Target-letter interaction was significant in the subject only (Figure 4), *F*1(4,152) = 3.95, *p* < 0.004, η^2^_p_ = 0.09, *F*2(4,120) = 1.75, *p* < 0.09, η^2^_p_ = 0.06; a Fisher’s LSD *post hoc* test (Bonferroni’s adjusted α level for significance, *p* < 0.0003) revealed that preservation ICs were more frequent on the third letter than the second letter in the case of the low-rise and high-rise SPs (e.g., L in IVLyde was misperceived as IVlyde more often than V in Ivlyde was misperceived as IVlyde), while violation ICs were more frequent on the second letter than the third letter in the case of the low-rise and high-rise SPs (e.g., V in IVlyde was misperceived as Ivlyde more often than l in IVlyde was misperceived as IVLyde). The Condition × Time × Group interaction was also significant (Figure 5), *F*1(2,76) = 14.77, *p* < 0.0001, η^2^_p_ = 0.28, *F*2(2,240) = 6.15, *p* < 0.003, η^2^_p_ = 0.05; a Fisher’s LSD *post hoc* test (Bonferroni’s adjusted α level for significance, *p* < 0.0008) confirmed that preservation ICs progressively increased from T1 to T3, while violation ICs decreased progressively from T1 to T3. Both response patterns progressed faster and more steeply in “good” readers than in “poor” readers, in particular for the violation ICs. However, preservation ICs were more important than violation ICs in T2 and T3 in good readers (*p*_*s*_ < 0.0001), and only in T3 in poor readers (*p* < 0.0003; marginally significant in T2, *p* < 0.08). In T1, differences were not significant (in good readers, *p* < 0.06; in poor readers, *p* < 0.09). The Sonority × Group interaction was not significant, *F*_*s*_ < 1.

### *Post hoc* Tests

Although we controlled *a priori* for some statistical and linguistic parameters, we assessed *a posteriori* the robustness of phonological sonority-based markedness. In doing so, we attempted to determine whether or not the syllable effects resulted from low-level similarities in gestural, spectral or acoustic-phonetic contrasts (e.g., Stevens, 2002). Our aim was not to determine what constitutes sonority parameters or what determines SPs. Rather, we carried out Tukey’s HSD *post hoc* tests on ten parameters based on manner-of-articulation parameters (tests 1–6), place-of-articulation parameters (test 7), letter confusability (test 8), and statistical properties (tests 9–10). We extended our analyses in order to ensure that our sonority-based effects (i.e., global sensitivity to SPs and syllable segmentation within the C_1_C_2_ clusters; ICs in%) did not depend on isolated parameters that are known to influence the location of the syllable boundaries. We ran the *post hoc* tests on the IC rate. All the *p*-values refer to the ICs collapsed for both conditions (i.e., color-syllable compatibility and color-syllable incompatibility) since none of the parameters that we tested interacted with Condition.

None of the distinctiveness due to any of the manner-of-articulation parameters had a significant effect:

(1) Voicing (i.e., voiced-voiced C_1_C_2_ clusters; *M* = 16.7% vs. voiceless-voiceless C_1_C_2_ clusters; *M* = 15.1%; *p* > 0.1)^6^;
(2) Sonorant (i.e., we performed a comparison between C_1_C_2_ clusters with two sonorant consonants; *M* = 16.2% and C_1_C_2_ clusters with two non-sonorant consonants; *M* = 16.6%; *p*_*s*_ > 0.1);
(3) Continuancy (i.e., we performed a comparison between C_1_C_2_ clusters with two non-continuant consonants; *M* = 15.9%, C_1_C_2_ clusters with two continuant consonants; *M* = 17.2%, and C_1_C_2_ clusters with a non-continuant and a continuant consonant, irrespective of whether they were in C_1_ or C_2_ position; *M* = 16.1%; *p*_*s*_ > 0.1);
(4) Stridency (i.e., we performed a comparison between C_1_C_2_ clusters with two strident consonants; *M* = 17.5% and C_1_C_2_ clusters with two non-strident consonants; *M* = 15.7%; *p* > 0.1);
(5) Nasality (i.e., we performed a comparison between C_1_C_2_ clusters with one nasal consonant in either the C_1_ or C_2_ position; *M* = 15.7%, and C_1_C_2_ clusters with two non-nasal consonants; *M* = 16.7%; *p* > 0.1);
(6) Manner similarity or dissimilarity (i.e., we performed a comparison between C_1_C_2_ clusters with two consonants with the same manner-of-articulation, irrespective of the similarity between manners; *M* = 17.0%, and C_1_C_2_ clusters with two consonants with a different manner-of-articulation, irrespective of the dissimilarity between them; *M* = 16.0%; *p* > 0.1). Similarly,
(7) The distinctiveness due to the place-of-articulation parameter did not have significant effects. We compared the direction within the C_1_C_2_ clusters (i.e., *forward* from anterior to posterior regions; *M* = 16.4%, and *backward* from posterior to anterior regions; *M* = 16.2%; *p* > 0.1). Also, (8) letter confusability did not produce any significant difference (low; *M* = 16.6%, and high; *M* = 16.0%; *p* > 0.1; i.e., DRC Letter Confusability Calculator^7^).

We next performed an analysis on (9) the frequency distance between the bigram that preceded, straddled and followed the syllable boundary. Although we tried to harmonize the sublexical frequencies, some distances varied within the pseudowords. However, no difference reached the threshold of statistical significance (e.g., null or quasi-null distance; *M* = 16.5%, and low distance *M* = 16.2%; *p* > 0.1). Finally, (10) the phonotactic transitional probabilities were calculated for the C_1_C_2_ clusters (Crouzet, 2000) and no difference reached the threshold of statistical significance (e.g., low; *M* = 16.0%, and high; *M* = 17.0%; *p* > 0.1).

## Discussion

The present study investigated whether – and how – universal phonological sonority-based markedness – through SP across syllable boundaries – drives syllable location and segmentation in visual identification in French children when the statistical properties of the orthographic and phonological constituents are null or quasi-null. To do this, we used the classical IC paradigm with beginning readers, who were subdivided into “good” and “poor” readers. To our knowledge, this is the first time that a short-term longitudinal study has used the IC paradigm to track developmental changes in syllable-based strategies with regard to reading level, phonological universals, and statistical parameters. We thus showed that syllable location and segmentation was possible without any statistical and distributional cue by deriving and applying phonological sonority-based markedness constraints to unattested intervocalic C_1_C_2_ clusters.

Our first results indicated non-statistical syllable segmentation strategies. Our results revealed that the absence of either orthographic or phonological statistical cues did not prevent the reliable location of the syllable boundaries. Contrary to the classical view, our results provide clear-cut evidence that syllable segmentation does not necessarily require the reading units to be defined by both orthographic and phonological information, i.e., that orthographic and syllabic boundaries coincide and that the absence of a bigram trough does not suppress or eliminate syllable segmentation effects (e.g., Doignon and Zagar, 2006; Doignon-Camus and Zagar, 2014). Combined with the fact that the IC paradigm does not tap lexical access, suppresses lexical competition and limits the top-down lexical process, the counter-intuitive response patterns, with more preservation ICs than violation ICs being found in the absence or quasi-absence of statistical information, therefore suggest that segmentation strategies do not necessarily occur as a by-product of statistical properties. Of course, this does not mean that beginning readers are unable to learn statistical distribution through repeated exposure to explicit orthographic information that is mapped to implicit phonological information. Although literature has confirmed that syllable effects appear early and are regularly reinforced through the addition of orthographic and phonological constituents that have not been previously encountered, here the use of the IC paradigm does not encourage the statistical learning of our unattested C_1_C_2_ clusters (e.g., Doignon-Camus and Zagar, 2014).

When reading levels were examined, we confirmed well-documented data about the progressive and *“reading level-dependent”* nature of syllable segmentation in French (early and sustainable sensitivity to syllables to segment and access words during learning to read; e.g., Colé et al., 1999; Doignon and Zagar, 2006). The new, informative finding that we obtained relates to the time at which syllable segmentation becomes dominant and outranks other strategies. Syllables were not activated at T1 in either the good or the poor readers, even though preservation ICs exceeded violation ICs to a greater extent in good readers than in poor readers. This first observation is consistent with the fact grapho-syllabic processing is not fully specified. By contrast, preservation ICs systematically exceeded violation ICs in the good readers at T2 and T3, while this was not the case until T3 in the poor readers. This response pattern is confirmed by the β criterion, which varied over time and progressed faster and more strongly from the absence of syllable segmentation toward syllable segmentation in the good readers than it did in the poor readers.

How, then, when the grapho-syllabic processing becomes available do beginning readers locate the syllable boundaries if neither orthographic nor syllable frequency explicitly modulates syllable access and the bigram trough does not cue syllable segmentation (e.g., Doignon and Zagar, 2006; Maïonchi-Pino et al., 2010)? Although we cannot rule out other unspecified, and even more complex statistical or featural properties (e.g., Hayes and Wilson, 2008; Albright, 2009), syllable segmentation might be the consequence of an early, fast and accelerated facilitatory activation of the connection between the orthographic and phonological syllables through a limited set of syllable structures and their neighbors (e.g., Mathey et al., 2006 for arguments on syllable activation in visual word recognition) since V and VC structures represent 10% of syllable structures (e.g., Léon, 2007). Though acceptable, this proposal seems to be insufficient to account cogently for the segmentation strategies.

The true answer seems to lie in children’s early, constant, and automatic sensitivity to phonological sonority-based markedness (e.g., Berent et al., 2011, 2012b; Gómez et al., 2014). Their response patterns indicate that syllable segmentation respects the well-formedness of the C_1_C_2_ clusters within the syllable boundaries. As markedness progressively increases from high-fall SPs (e.g., *lb*), i.e., the unmarked and most well-formed intervocalic clusters, to marked high-rise SPs (e.g., *gm*), i.e., the marked and most ill-formed intervocalic clusters, there is a gradual switch from preservation ICs to violation ICs. These response patterns indicate that preservation ICs automatically and gradually increase as the C_1_C_2_ clusters tend toward an optimal syllable contact (e.g., Vennemann, 1988; Gouskova, 2004). If splitting or grouping C_1_and C_2_ depends on sonority-based markedness, then the response patterns reveal that French children do not need to strictly adhere to V_1_C_1_.C_2_V_2_C_3_V_3_ segmentation. The non-negligible, gradual inverse patterns for the violation ICs are also consistent with the sonority-based markedness of C_1_C_2_ onset clusters. Indeed, sonority-based ill-formedness (high-rise SPs) within syllable boundaries reflects the optimal sonority-based well-formedness of onset clusters (e.g., Clements, 2006). And the children optimize the segmentation to satisfy the well-formedness induced by the sonority-based markedness of onset clusters (e.g., V_1_.C_1_C_2_V_2_C_3_V_3_ segmentation). This lends support to a linguistic rule that generally applies to French segmentation with attested ill- and well-formed C_1_C_2_ clusters: the *Maximum Onset Principle* (e.g., Kahn, 1976). Indeed, when a C_1_C_2_ cluster violates the principle of optimal syllable contact, the adopted segmentation strategies conform to language-specific constraints which might be based in sonority, i.e., the maximization of consonants in onset clusters in cases where the native phonological regularities permit C_1_C_2_ onset clusters (e.g., Kahn, 1976; Clements, 1990; Ettlinger et al., 2012). Indeed violation ICs with the Target-letter in second position increased with low- and high-rise SPs, which are optimal onset clusters, while preservation ICs with the Target-letter in third position increased with low- and high fall SPs, which are optimal syllable contacts. In other words, this implies the use of syllable segmentation strategies in which a high-fall SP (e.g., *“rj”*) is considered as phonotactically illegal and disallowed in word-initial position, with segmentation therefore occurring between C_1_ and C_2_, while a high-rise SP (e.g., *“jr”*) is considered as phonotactically legal and allowed in word-initial position, with the result that segmentation occurs between V and C_1_.

The observed response patterns also raise questions about the contribution of linguistic experience and language-specific knowledge. Indeed, both distinct sonority-based markedness patterns occurred even though the children did not learn – explicitly or implicitly – any orthographic or phonological information from unattested ill- and well-formed C_1_C_2_ clusters, either in onset position or within syllable boundaries. What is clear-cut is how French children process C_1_C_2_ clusters. Their knowledge is not restricted to language-specific orthographic and phonological patterns and they do not need extensive exposure to written material in order to be sensitive to sonority-based markedness. They are able to automatically derive universal sonority-based information independently of statistical information (provided through repeated exposures to oral and written material) and this helps them use the appropriate syllable segmentation strategies. This finding might therefore strengthen the hypothesis that humans “are sensitive to putatively universal restrictions on syllable structures” (p. 5839; Gómez et al., 2014). However, we did not learn as much about the time course of sonority-based markedness during the reading processes.

Another interpretation might be that children derive some abstract phonological features that are shared with attested C_1_C_2_ clusters in French (see the *Sonority Projection* effects found in speech perception and production; e.g., Berent et al., 2007; Daland et al., 2011; see also Hayes and Wilson, 2008)^8^. Furthermore, our *a posteriori* analyses confirm that none of the IC response patterns, i.e., preservation or violation, is directly due to statistical properties or sonority-unrelated features (i.e., manner- and place-of-articulation). Although we did not probe all the phonetic transformations that do exist in speech perception (e.g., schwa deletion, consonant lenition, etc.; e.g., Hayes and Steriade, 2004; Kirchner, 2004; Davidson, 2006, 2011; Bürki et al., 2011; Davidson and Shaw, 2012), we propose that the color misperception does not stem from spectral or acoustic-phonetic failures to encode and decode the C_1_C_2_ clusters, and is more abstract, since there is no obvious evidence that such cues are required and involved in reading strategies (e.g., Berent and Lennertz, 2010; Tamási and Berent, 2014). Furthermore, the IC patterns are not due to a gestural failure to articulate the C_1_C_2_ clusters, since there is no reliable evidence that children articulate or need to articulate them (e.g., Wright, 2004; Redford, 2008).

## Conclusion

Taken together, our results provide new and valuable information about both the fine-grained short-term developmental changes in the use of syllable segmentation strategies in silent reading in French children. Children are sensitive to universal phonological sonority-based markedness for the quick and efficient location of syllable boundaries in the absence or quasi-absence of statistical cues. They thus rely on sonority-based markedness to perform a bidirectional and gradual analysis that reveals the syllabification of compliant C_1_C_2_ clusters with optimal syllable contact. They also perform a resyllabification that favors onset clusters over coda clusters in the case of compliant C_1_C_2_ clusters with the Sonority Sequencing Principle lying within the syllable boundaries. This also indicates that the activation of phonological sonority-based markedness, which makes it possible to locate and discriminate the syllable boundaries, is automatic and non-suppressible, and might contribute to reading acquisition. More importantly, the sensitivity to sonority-based markedness does not depend on reading abilities in contrary to the use of syllable segmentation strategies. Poor readers were sensitive to SPs in the same way as good readers, suggesting this is an unimpaired – and not delayed – phonological competence (e.g., for similar conclusions in developmental dyslexia, see Berent et al., 2012, 2013, 2016; Maïonchi-Pino et al., 2012a, b). Despite the abundant literature suggesting its importance, the phonological and/or orthographic statistical distribution does not seem to be a requirement – even in beginning readers – for learning how to use syllables and therefore for locating syllable boundaries in French (e.g., Doignon-Camus and Zagar, 2014; Mahé et al., 2014). However, the progressive crossover between the two *extreme* sonority-based markedness SPs (i.e., high-fall and high-rise SPs), which leads to two distinct IC patterns, corroborates the need of a maximal sonority distance between the phonemes to describe optimal clusters and to underpin syllable segmentation (e.g., Selkirk, 1984; Marouby-Terriou and Denhière, 2002). While syllable segmentation is an early, quickly activated but progressive procedure that appears earlier in good than in poor readers, the response patterns rule out a strict left-to-right effect that might stem from orthographic letter-by-letter processing or phonological grapho-phonemic processing that supports phonological grapho-syllabic processing. Indeed, the detection of the target-letter does not depend on its position, C_1_ or C_2_, but on the sonority-based markedness of the C_1_C_2_ clusters (e.g., Maïonchi-Pino et al., 2012a, b, 2013; but see Sprenger-Charolles and Siegel, 1997). Finally, we provided further evidence in support of the idea that the IC paradigm, which possesses intrinsic strong cognitive-perceptual constraints, can be used with beginning readers to assess phonological universal and language-specific abilities (e.g., Doignon-Camus et al., 2013; Doignon-Camus and Zagar, 2014).

## Data Availability Statement

The datasets generated for this study are available on request to the corresponding author.

## Ethics Statement

This study was carried out in accordance with the recommendations of the Regional School Management Office and the Regional Ethics Committee (Auvergne-Rhône-Alpes). All the children participated after their parents had completed and signed an informed consent form. The experiment referenced in the present manuscript followed all the ethical standards of experimental and developmental psychology in children.

## Author Contributions

NM-P, AC, and MT designed the study. AC, MT, OL, and VL collected the data. NM-P analyzed the data and wrote the first draft of the manuscript. AC, MT, OL, VL, and LF equally contributed to read, comment, and approve the submitted manuscript.

## Conflict of Interest

The authors declare that the research was conducted in the absence of any commercial or financial relationships that could be construed as a potential conflict of interest.

## Footnotes

1 The use of 1.5 and 4.5 is based on a suggestion by Parker (2002), although the relative distance between 1 and 1.5 and 4 and 4.5 may not be equivalent. Glides are not represented, in particular since this would not be useful for our study in French.

2 These restrictions might not be expressed phonetically but rather as algebraic optimizational rules that guide the well-formedness of syllable patterns (e.g., Berent, 2013).

3 The Manulex and Manulex-infra databases provide the distributional lexical and sublexical frequencies for French elementary-school readers (from first to fifth grade).

4 All the stimuli were pre-tested with 16 French second-graders since we used pseudowords such as *umzude* and *ymdyve*, the first two first letters of which may be pronounced/ym/and/im/or/um/,/ↄ m/, or/$\tilde{\varepsilon }$/. In a reading aloud task, we found that the children mostly pronounced *um* as/ym/(98.4%) and *ym* as/im/(93.8%).

5 Consonants that share the same place of articulation and are considered to be more complex than heterorganic consonants at both the phonetic and articulatory levels, and that are also more likely to lead to compensation for coarticulation as well as to the delaying or mistiming of the articulatory output due to a greater gestural overlap (e.g., Jakielski, 2002).

6 It should be remembered that we *a priori* excluded C_1_C_2_ clusters with voicing differences in order to avoid progressive and regressive voicing assimilation.

7 www.cogsci.mq.edu.au/∼ssaunder/utils/lc.php

8 We would like to thank one of the reviewers who suggested referring to the *Substance Free Phonology* to discuss our results (e.g., for an in-depth presentation of the framework, see Reiss, 2017). Although the Substance Free Phonology might be relevant since it discusses the constraints implemented in the Optimality Theory (e.g., the markedness constraints), it rejects the concept of gradient for grammaticality (which was considered for the sonority scale that we used; e.g., Gouskova, 2004). Another argument lies in the fact that Substance Free Phonology does not consider underlying ill-formedness of segments; such segments just won’t be formed since they do not exist as a surface form of a language (i.e., output).

## Appendix

**TABLE:** Stimuli used in the experiment as a function of their sonority profile.

**High-fall SP**	**Low-fall SP**	**Plateau SP**	**Low-rise SP**	**High-rise SP**
ulbyre	ozbyge	okpyce	ydvyme	ybnyje
olvyde	ifkute	itpyfe	ugjybe	ymryje
urzyve	yftyke	ydgoze	ikfuce	yzryve
olgoze	ymjuze	ufsyke	otfuke	ivlyde
ymdyve	umzyde	ipkute	ulryge	ogmuze
umgove	uvgoze	ovzule	uzmube	udmube
urjyde	izgove	itkufe	ivnyze	ojryve

## Abbreviations

C: consonant
IC: illusory conjunction
SP: sonority profile
V: vowel.
